# Low Vision Aids Provision for Visually Impaired Egyptian Patients – A Clinical Outcome

**DOI:** 10.4103/0974-9233.48865

**Published:** 2009

**Authors:** Sherin Shaaban, Ahmad Rashid El-Lakkany, Ashraf Swelam, Ghada Anwar

**Affiliations:** 1 From the Department of Ophthalmology, Mansoura Ophthalmic Center, Mansoura University, El-Mansoura city, 35516, Egypt; 2 From the Department of Ophthalmology, Okayama University Graduate School of Medicine, Dentistry, and Pharmaceutical Sciences, Okayama City, Japan

**Keywords:** Low Vision aids, Visual Impairment, Visual Rehabilitation

## Abstract

**Purpose:**

To evaluate a low vision rehabilitation service implemented for heterogeneously diverse group of Egyptianpatients with vision loss in terms of improving their visual performance and fulfilling their visual needs.

**Methods:**

Fifty patients with low vision were included in a prospective study. History taking, ophthalmic examinationand evaluation of the visual functions were performed for all patients. The required magnification was calculated, andsubsequently a low vision aid was chosen after counseling with patients. Low vision aids were tried in office, followedby a period of training before patients received their own low vision aids. Follow up was done for 6 months.

**Results:**

All patients who were referred to the low vision unit were not satisfied with their current spectacles or lowvision aids. After training and prescription of suitable LVAs, the improvement in distance and near visual acuity wasstatistically significant (p<0.001). Fifty-six per cent of the patients (n=28) showed improvement in distance visualacuity of 5 lines or more, and 57% of the patients (n=27) could discern N8 print size or better. The most commonlyused aids were high powered near adds. Despite the complaints about the appearance and use of LVAs, 76% of thepatients reported being moderately to highly-satisfied with their aids.

**Conclusions:**

The significant improvement in the visual performance of patients with low vision after the prescriptionand training on the use of LVAs, associated with patients' satisfaction, confirms the importance of expanding lowvision rehabilitative services and increasing the public awareness of its existence and benefits.

The increasing numbers of patients who are old or visually impaired and who can no longer be helped by conventional optical, medical or surgical methods, represent a challenge to optometrists and ophthalmologists both in developed and developing countries.^1^ To date no definitive treatment for the common causes of visual impairment such as age-related macular degeneration (AMD), diabetic retinopathy or glaucoma have proven effective in stopping the deteriorating effect of those diseases on vision.^2^,^3^,^4^ There is significant individual, public health and community consequences of reduced vision, such as increased cost of education, reduced personal income and loss of productivity for those caring for the visually impaired.^5^

The most effective way to reduce the degree of handicap associated with visual impairment is to provide low vision aids (LVAs) as a part of a comprehensive low vision rehabilitative service.^6^ When dispensed appropriately, these simple magnifying devices can enhance residual vision and permit people with impaired sight to perform daily tasks such as reading.^3^,^7^ The success of low vision services depends on extending the job of a low vision provider from simply prescribing LVAs, into counseling and training low vision patients.^6^,^8^

In a developing country like Egypt, provision of low vision services represents a challenge due to the lack of knowledge of some of the health care providers of the existence of such services. Furthermore, Egypt lacks an effective national health insurance program that can cover the relatively high cost of LVAs.

The aim of this study was to evaluate the effectiveness of LVAs in improving both distance and near vision among 50 Egyptian patients of diversified etiology for low vision. We further aimed at evaluating the level of patients' satisfaction as well as at identifying the common complaints reported after use of LVAs.

## SUBJECTS AND METHODS

### Patients

Patients included in this study were selected at random from patients attending the low vision clinic of Mansoura Ophthalmic Center, Mansoura University, Egypt. Patients were included in the study if they had a best corrected visual acuity (BCVA) of less than 6/18 in the better eye; in accordance with WHO definition of low vision.^9^

Exclusion criteria were age less than 6 years, mental handicap, media opacity, illiteracy or visual acuity better than 6/18 or worse than 1/60. An informed consent was obtained from adults or parents of children enrolled in the study after detailed explanation of the nature and possible outcome of the study. The study conformed to the Declaration of Helsinki and was approved by the Research Ethical Committee of Mansoura University. The age of the 50 patients enrolled in this study ranged from 6 years to 88 years. Thirty-four patients were males (68%) while 16 were females (32%). Demographic data are summarized in Table 1.

**Table 1:** Biographical Characteristics of Patients Enrolled in Study

Category	n	%
Age (years)		
6 to 20	14	28
21 to 40	9	18
41 to 60	16	32
61 to 80	8	16
more than 80	3	6

Sex		
male	34	68
female	16	32

Refractive Error in Diopters*		
0.5-2.0	22	44
2.25-5.0	11	22
5.25-8.0	9	18
8.25-11.0	2	4
>11.0	6	12

* Spherical equivalent of the refractive error in diopters.

### Methods:

All patients underwent full history taking including patient's visual requirements and previous low vision evaluation or use of LVAs. Full ophthalmic examination was performed including visual acuity (VA) testing. Distant VA was measured unilaterally then bilaterally at 3 meters (using hand-held Feinbloom Distance test Chart, Designs for Vision Inc., Ronkoma, NY) and near visual acuity was then tested (using Keeler A series letter and Landolt test charts progressing from A20 down to A10, and using printed Arabic texts in different Times New Roman fonts {Point notation}: N6, N8, N10, N24, N32 and N48). Near visual acuity was measured binocularly at the patient's preferred distance and then at 25 cm using a +4.00 D reading add. Refraction was measured using streak retinoscopy when possible; otherwise a bracketing technique in the form of a trial of high powered spherical and cylindrical lenses was adopted. Central visual field was tested using Amsler Grid.

Before proceeding to the choice and training in the use of LVAs, a thorough discussion with the patient was performed to assess the patient's visual needs, to describe the nature of visual impairment and to explain its influence on visual performance, including limitations even after use of LVAs.

According to patient's needs, the required magnification was calculated. Magnification for distance was calculated using the formula: Magnification required= Required VA/ Present VA. For the near magnification, when Keeler A system was used, magnification was calculated using the formula: Magnification= 1.25^n^ (n= the number of steps of improvement required). When the N-point notation was used, the magnification was calculated as: Magnification required= Present VA/ Required VA.

In-office trials of variable LVAs were then started. For distant tasks, the available low vision aids were hand-held or spectacle-mounted telescopes, either in a fixed focus or variable focus form. For near tasks, microscopes, hand-held or stand magnifiers were offered to the patients. Non-optical aids such as reading stands, typoscopes, direct illumination or large print material were recommended according to each individual case. Patients were advised on how to use the aids and were individually trained on using different techniques such as steady eye strategy, eccentric fixation, focusing and tracking. After allowing patients to try variable aids, counselling to determine the suitable aid for each patient was performed, considering the needs, visual impairment status, and any other variables such as socio-economical factors. After the initial visit, 3 more in-office training sessions were performed. Each session was almost 30 minutes long. Patients were then allowed to purchase their own aids. The optical low vision aids used in this study were:

- Keeler Vision Enhancement Assessment Set.
- Schweizer Optik hand-held aspheric magnifiers, series 1840, Germany.
- Raylite, Coil illuminated stand-magnifiers, series 2, England.
- Coil half-eye microscopes with a built in base-in prism, England.

In-office follow up visits were planned up to 6 months, at the end of which an interview questionnaire was performed by the LVA therapist. Patients were asked about the frequency of use of LVAs, the duration of use each time, how difficult was it to use the aid after the in-office training, and the kind of complaints patients had while using the LVAs. Patients were also asked to rate their level of satisfaction with their LVAs and with the rehabilitation service in general.

## RESULTS

According to etiology of low vision, patients fell into 4 groups: Group A: patients with low vision attributed to a macular lesion; group B: patients with low vision attributed to optic atrophy; group C: patients who had both macular and optic nerve disease and group D: patients with low vision due to other causes. The etiology of low vision among patients enrolled in this study is summarized in Table 2.

**Table 2:** Etiology of Low Vision among the 50 Low Vision Patients

Disease	n	%
High myopia	13	26

Optic atrophy	7	14

Retinitis Pigmentosa	4	8

Stargardt's Disease	4	8

Diabetic Retinopathy	4	8

Rod-Cone Dystrophy	3	6

ARMD*	3	6

Nystagmus	3	6

Best' Disease	2	4

SRNM§	2	4

Macular Scar	2	4

Vein occlusion	1	2

Albinism	1	2

Aniridia	1	2

* age-related macular degeneration;

§ subretinal neovascualr membrane

At the time of presentation all patients were no longer satisfied with their present spectacles or LVAs if they were using any. The refractive errors of patients are represented as spherical equivalent and summarized in Table 1.

In accordance with the WHO categories of visual loss, thirty-two patients (64%) were visually impaired-BCVA worse than 6/18, but better than 6/60-, 8 patients (16%) were severely visually impaired-BCVA worse than 6/60, but better than 3/60-, while 10 patients (20%) were legally blind (BCVA worse than 3/60).

Differences in near VA between Keeler system and point system were observed, so we chose to report the results in Point system as it was in Arabic language and as a continuous text, while Keeler A system was in the form of Landolt's broken rings and as isolated symbols which might cause false high results. We did not include the results of 3 children who were considered non-proficient readers. At time of presentation only 3 patients (6%) could discern N8 print, 8 patients (17%) could discern N10 print, 5 patients (11%) could discern N24 print, 8 patients (17%) could discern N32 print, and 23 patients (49%) could only discern N48 print or even larger fonts.

**Figure 1:**
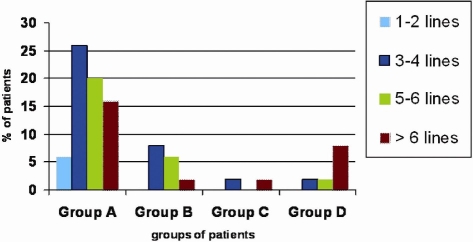
The improvement in distance visual acuity in terms of increase of number of lines visualized on Feinbloom Chart among the 4 etiological groups of patients. Group A: macular lesions, Group B: Optic nerve lesions, Group C: Both macular and optic nerve lesions and group D: Other causes. Fifty-six per cent of the patients showed improvement of 5 lines or more using telescopes.

Improvement of distance VA using telescopes showed statistical significance (Wilcoxon signed rank test, p<0.001). Twenty-eight patients (56%) showed improvement of 5 lines or more. Nineteen patients (38%) showed improvement of 3-4 lines and 3 patients (6%) showed mild improvement of 1-2 lines. The improvement in the groups according to the etiology is described in Figure 1. Of the 28 patients who showed an improvement of 5 lines or more, 64% belonged to group A, 14% belonged to group B, 4% belonged to group C and 18 % belonged to group D.

**Figure 2:**
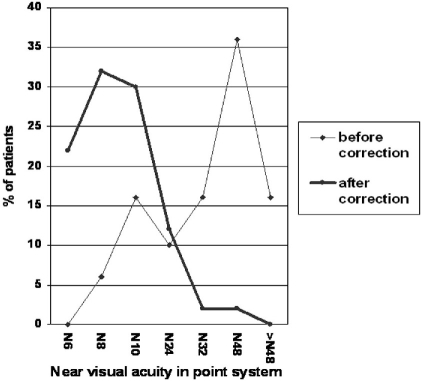
Near visual acuity levels, in Point system, before and after the use of low vision aids. Using low vision aids was associated with a marked increase in number of patients who could discern N8 point font size or better, and a decrease in the number of patients who could only discern N48 point font size or larger fonts

After provision of low vision aids, there was a significant increase in the number of patients who could discern N8 print and better. Twenty seven patients (57%) could discern N8 print and better. Thirty-one patients (66%) at presentation could only discern N32 or larger print, this number markedly decreased to only 2 patients (4%) after use of LVAs. Results of improvement in near visual acuity are detailed in Table 3 and Figure 2. The overall improvement in near visual acuity was statistically significant (Wilcoxon signed rank test, p<0.001).

**Table 3:** Improvement in Near Visual Acuity after Use of LVAs in Point System

Near Visual	Before Correction		After Correction	
Acuity	n	%	n	%
N6	0	0	11	23

N8	3	6	16	34

N10	8	17	12	26

N24	5	11	6	13

N32	8	17	1	2

N48	15	32	1	2

> N48	8	17	0	0

A correlation between improvement in near VA and the pre-correction level of distance VA was observed. Using LVAs, twenty-two patients (68.75%) of the visual impairment group could read print size N8 or better; 2 patients (25%) of the severe visual impairment group could discern N8, while three patients (33.3%) of the blind group could discern N8 print. Therefore the best improvement was achieved in the group of patients that were in the visually impaired group.

Twenty-seven patients (54%) asked for an aid to help them in near tasks, 17 patients (34%) asked for aids to help in both near and distance tasks, while only 6 patients (12%) needed aids to help in distance tasks only. Patients asking for distance tasks only were children.

**Figure 3:**
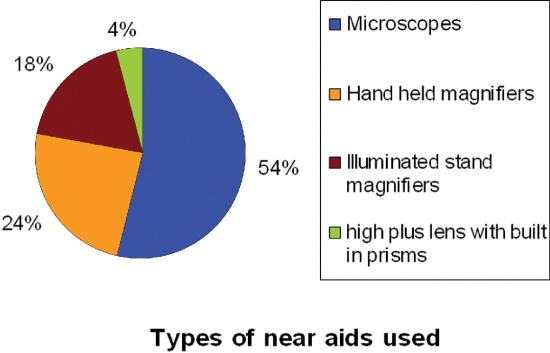
Distribution of LVAs prescribed for patients to help in near tasks such as reading. The most commonly prescribed near aids were microscopes followed by hand-held magnifiers

The magnification level of prescribed aids ranged between 2X and 10.1X. More than half of the patients (58%) used aids with a range of power between 2X and 5X, while 42% of the patients used aids ranging from 5.4X to 10.1X. High powered reading aids (microscopes) were the most commonly used near aid (54%), followed by hand-held magnifiers (24%). Figure 3 illustrates the types of near aids used by our patients.

Non-optical aids were prescribed for 32 patients (64%). The most common were the direct illumination and reading stands. Large print text was used to assist 3 patients (6%) who only needed to read The Holy Books which were the only commercially available texts in large print in Egypt.

**Figure 4:**
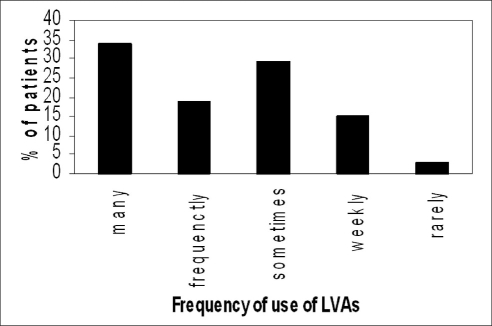
The frequency of use of LVAs according to response of patients when asked how often they used their aids. Many times= more than 10 times/day, frequently= 5-10 times/day, sometimes= 2-5 times/day, weekly= once or twice/week and rarely=once or twice/month

**Figure 5:**
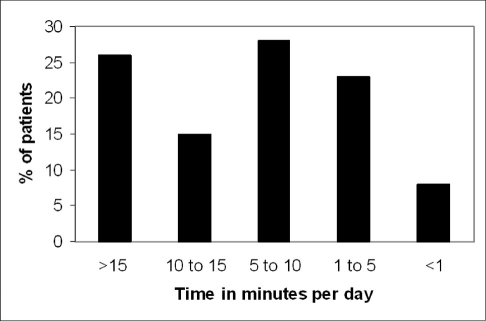
Distribution of response of patients about duration of use of LVAs in minutes per day. Five categories were identified: (1) more than 15 minutes/day, (2) 10-15 minutes/ day, (3) 5-10 minutes/day, (4) 1-5 minutes/day, (5) less than 1 minute/day

**Figure 6:**
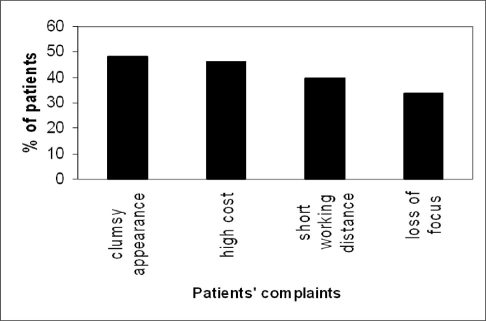
Most common complaints reported by the 50 patients after using LVAs. The clumsy appearance was the most annoying complaint (48% of patients), followed by the high cost (46% of patients).

Figures 4 and 5 summarize the patients' responses to questions about the frequency of use of the aid and duration of time of use of the LVAs per day. According to patients response to a question about the ease of use of the aid, 18 patients (36%) reported the aid being easy to use, 23 patients (46%) reported it a little difficult, 8 patients (16%) said it was very hard to use, although those patients reported being able to manage the difficulty with time. One patient (2%) reported that it was extremely difficult to use the aids alone, and hence additional in-office training sessions were planned. The most commonly reported complaints were the clumsy appearance of the aids, high cost, short working distance and loss of focus. Figure 6 illustrates the incidence of complaints among the 50 patients. When patients were asked if they would rate their satisfaction with their aids as highly, moderately or poorly satisfied, fifteen patients (30%) reported to be highly satisfied, 23 patients (46%) were moderately satisfied while 12 patients (24%) were poorly satisfied. As for the overall rehabilitation service, 34 patients (68%) mentioned that the service was very helpful, 13 patients (26%) reported it was helpful, while the remaining 3 patients (6%) reported being frustrated about the service.

## DISCUSSION

The findings of our study confirm that the provision of low vision aids is associated with a statistically significant improvement in both near and distance visual acuities and with patients' satisfaction.

Despite the fact that provision of low vision services prove to be associated with improved functional status and quality of life of patients with visual impairment,^2^,^10^ many of the patients or their health care providers are either unaware of the availability of such services or have no access to it.^11^,^12^,^13^,^14^ The problem is even worse in developing countries where low vision services are striving to exist,^5^,^16^ and where the economical situation affects the ability of both the health institutions and individuals who are visually impaired to afford such services. In light of the expected increase of numbers in the patients with visual impairment,^1^,^17^ and with the scarcity of studies evaluating the effectiveness of low vision aids,^2^,^4^,^18^ we aimed at quantifying the improvement in distance and near visual acuity after prescribing and training patients in the use of suitable low vision aids. We also aimed at identifying what kind of complaints could hinder the continuity of use of prescribed aids and how would the patients rate their satisfaction about the rehabilitation service.

Interestingly, unlike the epidemiological results of many studies,^8^,^19^,^20^ the mean age of our patients' sample was much less. We assume that the reason behind this is the fact that older patients in developing societies like Egypt live with other family members and depend on them much more. Such elderly patients become more reluctant to seek seemingly complicated aids that need special training and new adaptive techniques. Furthermore the illiteracy rate in developing societies is very high especially among older patients,^21^ while the need to read represents the major requirement for an age-matched group of patients from developed countries. The life expectancy is also less in developing countries.^16^ For these reasons we assume that those young, literate socially active patients in developing countries are mainly the ones who seek low vision services.

In accordance with this was the etiology of visual impairment among our sample. Sixty-eight per cent of the patients in this study had macular diseases, yet only 6% of those were due to age-related causes such as AMD, while the rest were mostly congenital in nature. This represents another point of difference compared to studies reported elsewhere.^19^,^20^,^22^

We observed that the improvement in distance visual acuity was not dependent on the underlying pathology, since the etiology profile of the patients showing improvement in distance visual acuity to 5 lines or more was almost identical to the etiology profile of the whole patients' sample. Similar findings were previously reported.^2^ On the other hand a correlation was observed between the improvement in near VA and pre-correction level of distance VA, where patients with a better level of pre-correction distance VA achieved a better post-correction near VA. Such an observation could be utilized as a success predictor when providing LVAs.

Analysis of the complaints of the patients after the use of aids in this study revealed that the clumsy appearance was the main complaint, especially when the patient started to use the aid in front of relatives, work or class mates. Another reason was the need to adopt new techniques for reading or using the LVAs with a sense of permanent loss of pre-visual impairment reading abilities, which was perceived as a declaration of patient's permanent handicap. Patients reporting to be frustrated about the service were mainly those who had unrealistic expectations even after counseling and discussion about the limitations of LVAs in terms of its functional as well as cosmetic aspects.

One limitation to the accurate assessment of the visual performance of patients in this study was the use of only the ability to read small print, without assessing neither the speed nor the duration of reading or performing the visual tasks. Another limitation was the inability of our institute to provide patients with trial closed-circuit televisions due to financial restrictions, as well as our assumption that our patients would not be able to afford such aids even if they prove effective.

This study is the first in Egypt to report the outcome of a low vision rehabilitation service. Relative to the costs of visual impairment, the provision of low vision rehabilitation services seem to be quite low. In a developing country such as Egypt, increased awareness of the public and the medical health providers of the availability and the benefits of such services is expected to help improve the quality of life of patients who are visually impaired. The concept that nothing further could be done for individuals who are visually impaired might be changed and perhaps health authorities might eventually be encouraged to finance such services.
